# Online Self-Presentation Strategies and Fulfillment of Psychological Needs of Chinese Sojourners in the United States

**DOI:** 10.3389/fpsyg.2020.586204

**Published:** 2021-01-29

**Authors:** Tian Yang, Qianwei Ying

**Affiliations:** ^1^School of Overseas Education, Sichuan University, Chengdu, China; ^2^Business School, Sichuan University, Chengdu, China

**Keywords:** self-presentation strategies, fulfillment of need for autonomy, fulfillment of need for relatedness, social media, Chinese sojourners

## Abstract

This study statistically analyzed survey data to examine the relationship between fulfillment of psychological needs of 223 Chinese sojourners in the United States and their online self-presentation strategies on Chinese and American social media. The results showed that the combined use of proactive and defensive self-presentation strategies on Chinese social media instead of American social media were more effective to fulfill the sojourners’ need for autonomy. Moreover, presentation strategies that helped to meet the sojourners’ need for relatedness were significantly different between Chinese and American social media. Specifically, a proactive strategy was more effective to meet sojourners’ need for relatedness on Chinese social media, while a defensive strategy was more effective to fulfill their need for relatedness on American social media.

## Introduction

Self-presentation is the core concept of American sociologist Irving Goffman’s *Dramaturgy*. As an individual’s role-playing behavior of self-expression in interpersonal interaction, self-presentation provides an impetus for self-promotion in real life (Goffman, 1959). Western social psychologists have tested and revised the Goffman’s theory (Jones and Nisbett, 1971), and the impression management theory (IMT) has been developed, which suggests that people apply a series of strategies (such as modification, concealment, and decoration) to control others’ perception of themselves as impression decoration or self-presentation.

With social media widely involved in people’s daily lives, there have appeared an increasing number of studies that are based on the theories of *Dramaturgy* and the self-determination theory (SDT), analyzing the relationship between online self-presentation behavior and the fulfillment of psychological needs. Online self-presentation is an important part of online social interaction and is influenced by multiple factors such as individual psychology, social context, and social culture. For instance, self-enhancers will selectively choose only positive life events and favorable personal information to share with their social network friends, but other people may entail presenting both positive and negative aspects of the self on social media to reveal their true feelings (Lee-Won et al., 2014; Bareket-Bojmel et al., 2016).

In terms of self-presentation and need for relatedness, for example, Deters and Mehl (2013) pointed out that the active self-presentation on Facebook can reduce loneliness; Pittman and Reich (2016) found that compared with text-based platforms, social media users’ presentation on image-based platforms significantly reduced loneliness due to their enhanced intimacy with others. In terms of self-presentation and the need for autonomy, since a more multidimensional space for self-determined behaviors is provided in social media (Reinecke et al., 2014), people can freely present their true selves without being affected by the outside world, therefore meeting their needs for autonomy (Chen, 2019). For immigrants or sojourners, studies have found that they are more inclined to fulfill their autonomy needs through self-presentation on ethnic social media (Mikulincer and Shaver, 2007; Lim and Pham, 2016; Pang, 2018; Hofhuis et al., 2019). Additionally, proactive self-presentation strategies were found to be positively related to the maintenance of psychological well-being (Swickert et al., 2002; Kim and Lee, 2011; Ellison et al., 2014; Stieger, 2019), and in order to obtain more social support, people need to keep a balance between the use of selective and authentic presentational strategies (Bayer et al., 2020).

The psychological effect of online self-presentation has attracted more and more academic attention. However, these studies still remain inconclusive as how people fulfill their psychological needs by means of online self-presentation behavior in intercultural contexts. Specifically, most studies of sojourners are conducted in unitary contexts, either in sojourners’ ethnic social media environments or the social media of the host country, ignoring sojourners’ co-performance in dual-cultural contexts. Moreover, with the growth of the scale of Chinese sojourners, an increasing number of studies have been aimed at them, yet most have focused more on acculturation problems than online self-presentation behaviors. However, online self-presentation has gradually become an important behavior mechanism for Chinese sojourners’ acculturation and communication under the increasing influence of social media. Therefore, it is necessary to fill in the gaps in current research has left and to investigate the logical relationship between the online self-presentation and fulfillment of psychological need of Chinese sojourners in China and America’s dual-cultural contexts.

As important members of intercultural communication groups, Chinese sojourners in the United States are in the dual-cultural contexts of Chinese and American social media, thus they are ideal research participants. In view of this, this study focuses on the following questions:

RQ 1: Do Chinese sojourners mainly use Chinese or American social media to fulfill their psychological needs?
RQ 2: What kinds of presentation strategies are more effective in fulfilling Chinese sojourners’ psychological needs in dual-cultural contexts?

The purpose of this research is to study the logical relationship between online self-presentation strategies and the fulfillment of psychological needs (for autonomy and relatedness) of Chinese sojourners in the context of American and Chinese cultures and to further understand the characteristics of the psychological effects of Chinese sojourners’ online self-presentation behavior in intercultural contexts, so as to provide a new and resourceful way of thinking about maintaining Chinese sojourners’ mental health, as well as helping them to acculturate and communicate more effectively.

## Methods

### Participants and Procedure

This study focused on Chinese sojourners, who are mainly distributed on the east and west coasts of the United States. However, due to factors, such as the uniqueness of sojourners’ identity and their mobility, it is not possible to verify the official statistics on the population data. Therefore, the sampling method used in this study was a nonrandom sampling, and we were utilizing snowball sampling approach to recruit participants.

To be specific, our study initially chose Chinese overseas students, visiting scholars (college teachers and Confucius Institute teachers), and Chinese with a working visa in Washington state in the northwest of the United States as the main sample groups. We applied “Wenjuanxing” (wjx.cn), the most commonly used online questionnaire platform, to send out our questionnaires to people we knew in these three sample groups. We asked them to fill out the questionnaires and distributed the questionnaire link to their interpersonal social networks, including the WeChat groups of Chinese students studying in the United States and visiting scholars in American Colleges and universities, as well as online communities of local American Chinese. Following these procedures, we collected a snowballing sample of 300 questionnaires with responses.

In order to further reduce the error, the study carefully checked the responses to the 300 questionnaires; 29 questionnaires that did not indicate the use of both Chinese and American social media were excluded from the total sample, leaving 223 questionnaires as statistically valid. According to the data analysis of the demographic characteristics of the sample (see Table 1), a total of 135 female and 88 male sojourners participated in the survey. In terms of age, they ranged from 17 to 60 years of age, and the number of people aged between 21 and 30 was the biggest (120 people); there were 211 sojourners who had lived in the United States for 1 year or more.

**Table 1:** Descriptive statistics for demographic characteristics of participants.

Variables	Definitions	Number of participants	Mean	SD	Minimum	Median	Maximum
Gender	Gender of each participant, taking the value of 1 if the participant is male, and 0 otherwise	223	0.39	0.49	0	0	1
Age	The age of each participant	222	31.35	9.14	17	29	71
Marriage	Marriage status of each participant, taking the value of 1 if the participant if married and 0 otherwise	223	0.50	0.50	0	0	1
Education	Education level of each participant, taking the value of 1 for undergraduate education and below, 2 for master education, and 3 for doctoral education	223	2.17	0.75	1	2	3
Years	Years of stay in America	223	5.41	5.24	1	4	32
Time	Hours spent on social media daily, taking the value of 1 if less than half an hour, 2 if between half an hour to 1 h, 3 if between 1 h to 2 h, and 4 if more than 2 h.	223	2.82	0.95	1	3	4

Finally, based on the data collected, this study performed a descriptive statistical analysis of self-presentation strategies and psychological needs on Chinese and American social media followed by a regression analysis of the two main variables.

### Measures

Self-presentation strategies and the fulfillment of psychological needs were two major variables in our questionnaire, and both of them were measured with multiple items that were modified from established scales (Lee et al., 1999; Partala, 2011; Chen, 2019).

#### Self-Presentation Strategies

Although there were differences in the classification of self-presentation strategies in the field of psychology at the microlevel, the self-presentation strategies could still be divided into two categories: proactive strategies and defensive strategies (Goffman, 1959; Arkin et al., 1980; Tedeschi and Melburg, 1984; Fiske and Taylor, 1991). Based on this dichotomy and the self-presentation tactic scale developed by Lee et al. (1999), as well as our empirical observation of Chinese sojourners’ online self-presentation behavior in the United States, this paper specified six presentational tactics, namely “posting selected photos,” “expressing humorous and close content,” and “displaying discipline” for proactive strategies, aimed at actively shaping and maintaining an ideal image and, “expressing controlled feelings,” “self-taunting,” and “reporting only good news” for defensive strategies, aimed at preventing others from depreciating or belittling one’s image. These tactics were measured with six statements; responses were captured on a 5-point Likert scale from “1-never use” to “5-use almost every time.”

#### Psychological Need Fulfillment

Our measure of the fulfillment of need for autonomy was based on scale for the satisfaction of psychological needs on social networking sites developed by Partala (2011) and was specified with the statements “I feel that my choices express my ‘true self’” and “I have a say in what happens and can voice my opinion.” To measure the fulfillment of need for relatedness, we adapted existing measures of need satisfaction (La Guardia et al., 2000; Ryan et al., 2006; Partala, 2011) to the intercultural context on social media. Specifically, sojourners mainly maintained and developed three types of relationships in the intercultural context: the relationship with relatives and friends in their home country, the relationship with co-nationals or immigrants of the same cultural background, and the relationship with the locals in the host country (Lim and Pham, 2016; Hofhuis et al., 2019; Liu and Kramer, 2019). Based on the existing research, this study divided the needs for relatedness of Chinese sojourners in the United States into three categories: first, relational need with domestic relatives and friends, which was stated as “I feel close and connected with my domestic relatives and friends”; second, relational need with Chinese Americans, which was stated as “I feel a sense of contact with Chinese Americans”; third, relational need with Americans, which was stated as “I feel a sense of contact with Americans.” Responses were captured on a 5-point Likert scale from “1-totally disagree” to “5-totally agree.”

In order to understand the basic identity characteristics of Chinese sojourners, this study designed demographic characteristics variables, including “gender,” “age,” “marital status,” “education level,” “time to the United States,” “daily social media use time.” On this basis, this study designed a set of scale to evaluate the online self-presentation behavior of Chinese sojourners in the United States from the overall level. The scale consists of three parts: demographic information, self-presentation strategy, and psychological need fulfillment. Responses were captured with 5-point Likert scales, except for demographic characteristics. Since WeChat and Facebook were the two social media that are most frequently used according to our preliminary study on Chinese sojourners’ general use of social media, this paper chose WeChat and Facebook as the main platforms to observe and analyze the self-presentation behavior of the sojourners. On the basis of quantitative research, this study conducted interviews with 18 Chinese sojourners from all the respondents to understand the logical relationship between self-presentation strategies and fulfillment of psychological needs on Chinese and American social media.

## Results

### RQ 1: Do Chinese Sojourners Mainly Use Chinese or American Social Media to Fulfill Their Psychological Needs?

In order to answer this question, this study conducted a descriptive statistical analysis of the questionnaire data, and the results are shown in Table 2. We first calculated the average score of the respondents’ psychological needs on social media in China and the United States and then used a *t*-test to compare the difference of the average scores between Chinese and American social media. As for “the fulfillment of the need for autonomy,” the results showed that the average score of Chinese social media was significantly higher than that of American social media at the level of 1%, indicating that the self-presentation behavior of Chinese social media was more effective for the fulfillment of Chinese sojourners’ need for autonomy.

**Table 2:** Fulfillment of the needs for autonomy and relatedness in American and Chinese social media.

Variables	Statements	American social media	Chinese social media	Difference
Fulfillment of need for autonomy	I feel that my choices express “my true self”	3.128^*^	3.632^***^	−0.514^***^
	I have a say in what happens and can voice my opinion	2.835^**^	3.448^***^	−0.628^***^
Fulfillment of need for relatedness	I feel a sense of contact with Chinese Americans	2.913	3.520^***^	−0.619^***^
	I feel close and connected to my domestic relatives and friends		4.220^***^	
	I feel a sense of contact with Americans	3.321^***^		

* Indicates 10% significance.

** Indicates 5% significance.

*** Indicates 1% significance.

In terms of “the fulfillment of the need for relatedness,” the average score of American social media was significantly higher than 3 (a score of 3 represents neutrality), indicating that the development of a relationship with Americans through online self-presentation was significant. In Chinese social media, the average score of “maintaining the relationship with domestic relatives and friends” was significantly higher than 3 at the level of 1%, indicating that Chinese social media had a significant impact on the relationship with family and friends back in China. As for maintaining a relationship with American Chinese, the average score of Chinese social media was significantly higher than that of American social media at the level of 1%, suggesting that the Chinese social media could promote the relationship between sojourners and American Chinese more effectively than American social media.

### RQ 2: What Kinds of Presentation Strategies Are More Effective to Fulfill Chinese Sojourners’ Psychological Needs in the Dual-Cultural Contexts?

In order to test the relationship between online self-presentation strategies and the fulfillment of psychological needs, this study further applied a regression analysis after controlling the demographic characteristics of sojourners such as gender, age, marital status, education level, years in the United States, and time spent on social media. The specific regression model was as follows:

$$\begin{array}{l} Effect_{i}=\alpha +\beta_{1}Strategy_{i}+\beta_{2}Gender_{i}+\beta_{3}Age_{i}+\\ \beta_{4}Marriage_{i}+\beta_{5}Education_{i}+\beta_{6}Years_{i}+\beta_{7}Time_{i}+\varepsilon \end{array}$$

Among them, the dependent variable *Effect* represented the fulfillment of psychological needs (autonomy and relatedness) brought by the online self-presentation behaviors of the Chinese sojourners, and the independent variable *Strategy* represented the self-presentation strategies including “posting selected photos,” “expressing humorous and close content,” “displaying discipline,” “reporting only good news,” “expressing controlled feelings,” and “self-taunting.” The control variables included the sojourners’ gender (*Gender*), age (*Age*), marital status (*Marriage*), education level (*Education*), length of stay in America (*Years*), and hours spent on social media daily (*Time*). Table 1 illustrates the descriptive statistics for the above demographic characteristics of participants in our regression.

We have found in Table 2 that Chinese sojourners’ self-presentation behavior on Chinese social media is more effective in fulfilling their need for autonomy. Therefore, we conducted a regression analysis on the relationship between the presentation strategies adopted by the sojourners on Chinese social media and their need for autonomy (see Table 3 for the research results). It was found that all six presentation strategies can significantly promote the fulfillment of the sojourners’ need for autonomy but that there are differences in the effectiveness of these strategies. Specifically, for the autonomy dimension of “expressing one’s true self,” the strategy with the most obvious effect was the proactive strategy “expressing humorous and close content,” while for the autonomy dimension of “voicing one’s opinion,” the strategy with the most obvious effect was the defensive strategy “expressing one’s controlled feelings.” It could be seen that in the context of social media in China, the combination of proactive and defensive strategies played a more positive role in meeting the need for autonomy. Through offline interviews, the results of quantitative analysis were further supported. Interviewees have said that the presentation strategy of “expressing humorous and close content” played an important role in arousing emotional resonance and expressing one’s true self; while for important events in personal or social life, using the defensive strategy of “expressing one’s controlled feelings” was more helpful for sojourners to voice his or her opinion in an objective stand and build an intercultural image with the ability of reflection.

**Table 3:** The effect of different self-presentation strategies on the fulfillment of the need for autonomy in Chinese social media.

**Panel A. Measuring autonomy by asking “I feel that my choices express my true self”**						
Strategy choices:	Posting selected photos	Expressing humorous and close content	Displaying discipline	Reporting only good news	Expressing controlled feelings	Self-taunting
Strategy	0.247^***^	0.307^***^	0.171^**^	0.155^***^	0.276^***^	0.247^***^
	(4.39)	(4.24)	(2.50)	(2.64)	(4.57)	(3.71)
Gender	−0.092	−0.144	−0.131	−0.160	−0.071	−0.131
	(−0.75)	(−1.13)	(−0.99)	(−1.24)	(−0.56)	(−1.03)
Age	0.001	0.001	−0.003	−0.001	−0.000	0.003
	(0.14)	(0.14)	(−0.39)	(−0.13)	(−0.06)	(0.31)
Marriage	0.158	0.225	0.197	0.187	0.154	0.212
	(1.07)	(1.48)	(1.25)	(1.21)	(1.03)	(1.43)
Education	−0.075	−0.148^*^	−0.111	−0.116	−0.104	−0.119
	(−0.98)	(−1.87)	(−1.36)	(−1.49)	(−1.34)	(−1.56)
Years	−0.017	−0.025^*^	−0.022^*^	−0.020	−0.019	−0.029^**^
	(−1.40)	(−1.96)	(−1.70)	(−1.48)	(−1.64)	(−2.31)
Time	−0.007	0.021	0.052	0.047	0.036	0.027
	(−0.09)	(0.30)	(0.73)	(0.67)	(0.55)	(0.37)
Constant term	3.047^***^ (6.88)	3.021^***^ (6.08)	3.469^***^ (7.32)	3.480^***^ (8.17)	2.994^***^ (6.70)	3.190^***^ (7.62)
Samples	223	223	223	223	223	223
R-squared	0.145	0.139	0.085	0.088	0.157	0.121
Panel B. Measuring autonomy by asking “I have a say in what happens and voice my opinion”						
Strategy choices:	Posting selected photos	Expressing humorous and intimate content	Displaying discipline	Reporting only good news	Expressing controlled feelings	Self-taunting
Strategy	0.162^***^ (2.62)	0.153^**^ (2.15)	0.151^**^ (2.11)	0.109^*^ (1.83)	0.181^***^ (2.78)	0.155^**^ (2.12)
Gender	−0.038	−0.075	−0.055	−0.082	−0.024	−0.065
	(−0.27)	(−0.54)	(−0.39)	(−0.59)	(−0.17)	(−0.46)
Age	−0.006	−0.007	−0.009	−0.008	−0.007	−0.006
	(−0.62)	(−0.68)	(−0.91)	(−0.76)	(−0.75)	(−0.54)
Marriage	0.313^**^	0.357^**^	0.332^**^	0.330^**^	0.311^*^	0.349^**^
	(2.01)	(2.25)	(2.07)	(2.09)	(1.96)	(2.21)
Education	0.017	−0.026	−0.005	−0.009	−0.001	−0.011
	(0.19)	(−0.29)	(−0.05)	(−0.10)	(−0.01)	(−0.13)
Years	−0.006	−0.011	−0.008	−0.007	−0.007	−0.013
	(−0.40)	(−0.70)	(−0.55)	(−0.47)	(−0.50)	(−0.88)
Time	0.203^***^	0.228^***^	0.239^***^	0.238^***^	0.231^***^	0.226^***^
	(2.68)	(3.19)	(3.33)	(3.32)	(3.29)	(3.03)
Constant term	2.356^***^	2.487^***^	2.516^***^	2.613^***^	2.322^***^	2.471^***^
	(4.91)	(5.09)	(5.15)	(5.64)	(4.94)	(5.34)
Samples	223	223	223	223	223	223
R-squared	0.114	0.098	0.103	0.096	0.118	0.103

T statistics were calculated based on White robust standard errors are in parentheses.

* Indicates 10% significance.

** Indicates 5% significance.

*** Indicates 1% significance.

The empirical analysis in this paper had shown that the self-presentation behavior was effective in fulfilling sojourners’ need for relatedness in both Chinese and American social media. In order to investigate the differences between presentation strategies used in Chinese and American social media, this paper then conducted a regression analysis of the two platforms’ presentation strategies and fulfillment of sojourners’ needs for relatedness.

For Chinese social media, panel A in Table 4 shows that except “reporting only good news,” the other five presentation strategies have positive effects on maintaining the relationship between sojourners and their domestic relatives and friends. However, there were differences in the effectiveness of these strategies in fulfilling such a need, specifically, the proactive strategies of “expressing humorous and close content” and “displaying discipline” were comparatively more effective in fulfilling sojourners’ need to maintain domestic relationships. Similarly, the results in panel B shows that only the two proactive strategies of “displaying discipline” and “expressing humorous and close content” played an active role in maintaining the relationship between sojourners and Chinese Americans. It could be seen that the self-presentation on Chinese social media, whether to meet the relational needs with domestic relatives and friends or with Chinese Americans, was more effective by adopting proactive presentation strategies. The results of offline interviews further supported the quantitative research results. Interviewees said that “expressing humorous and close content” played an important role in maintaining the relationship with domestic relatives and friends, and this strategy could help them to narrow down the emotional distance with their relatives and friends back in China. At the same time, interviewees often expressed humorous and close content in the WeChat group of “Fellow Countrymen Association,” so as to promote the emotional connection with Chinese Americans. Also, interviewees considered as it necessary to present their “principled” side on Chinese social media and pointed out that “forwarding + commenting” was the most effective way to show the principle. Interviewees said that the strategy of “displaying principle” could help them to shape their self-image of self-discipline, self-reliance, and maintenance of their own cultural identity, thus strengthening the connection with their domestic relatives, friends, and Chinese Americans.

**Table 4:** The effect of different self-presentation strategies on the fulfillment of the need for relatedness in Chinese social media.

Panel A. Measuring relatedness by asking “I feel close and connected with my domestic relatives and friends”						
Strategy choices:	Posting selected photos	Expressing humorous and close content	Displaying discipline	Reporting only good news	Expressing controlled feelings	Self-taunting
Strategy	0.130^**^	0.230^***^	0.199^***^	0.023	0.089^*^	0.147^***^
	(2.57)	(3.44)	(3.55)	(0.47)	(1.78)	(2.74)
Gender	−0.157	−0.180^*^	−0.156	−0.196^*^	−0.166	−0.176
	(−1.40)	(−1.66)	(−1.41)	(−1.73)	(−1.44)	(−1.57)
Age	−0.007	−0.006	−0.010	−0.009	−0.008	−0.006
	(−0.79)	(−0.70)	(−1.12)	(−0.98)	(−0.92)	(−0.66)
Marriage	0.396^***^	0.432^***^	0.400^***^	0.426^***^	0.409^***^	0.424^***^
(married)	(2.92)	(3.23)	(2.98)	(3.07)	(2.98)	(3.12)
Education	0.081	0.036	0.066	0.059	0.063	0.058
	(1.25)	(0.56)	(1.05)	(0.92)	(0.98)	(0.95)
Years	−0.008	−0.012	−0.008	−0.011	−0.010	−0.014
	(−0.58)	(−0.89)	(−0.64)	(−0.78)	(−0.71)	(−1.05)
Online time	0.077	0.081	0.100^*^	0.111^**^	0.105^*^	0.092^*^
Per day	(1.33)	(1.55)	(1.84)	(2.02)	(1.87)	(1.69)
Constant items	3.427^***^	3.201^***^	3.328^***^	3.842^***^	3.599^***^	3.448^***^
	(8.57)	(9.01)	(9.05)	(10.12)	(9.49)	(9.47)
Samples	223	223	223	223	223	223
R-squared	0.127	0.157	0.155	0.095	0.108	0.126
Panel B. Measuring relatedness by asking “I feel a sense of contact with Chinese Americans”						
Strategy choices:	Posting selected photos	Expressing humorous and close content	Displaying discipline	Reporting only good news	Expressing controlled feelings	Self-taunting
Strategy	0.011	0.139^*^	0.184^**^	0.075	0.047	0.110
	(0.17)	(1.67)	(2.35)	(1.35)	(0.70)	(1.48)
Gender	0.088	0.095	0.123	0.088	0.101	0.101
	(0.65)	(0.69)	(0.89)	(0.64)	(0.76)	(0.74)
Age	−0.019^*^	−0.018	−0.020^*^	−0.019^*^	−0.019^*^	−0.017
	(−1.67)	(−1.60)	(−1.77)	(−1.66)	(−1.68)	(−1.54)
Marriage	0.431^**^	0.435^**^	0.405^**^	0.417^**^	0.423^**^	0.429^**^
	(2.48)	(2.50)	(2.33)	(2.43)	(2.40)	(2.49)
Education	−0.103	−0.118	−0.098	−0.104	−0.102	−0.105
	(−1.01)	(−1.21)	(−0.99)	(−1.04)	(−1.02)	(−1.07)
Years	0.006	0.006	0.009	0.008	0.007	0.004
	(0.39)	(0.39)	(0.57)	(0.49)	(0.44)	(0.26)
Online time	0.192^***^	0.176^**^	0.182^***^	0.187^***^	0.190^***^	0.179^**^
Per day	(2.68)	(2.53)	(2.67)	(2.75)	(2.77)	(2.48)
Constant	3.588^***^	3.195^***^	3.081^***^	3.386^***^	3.463^***^	3.277^***^
Term	(7.09)	(6.77)	(6.30)	(7.51)	(7.11)	(7.44)
Samples	223	223	223	223	223	223
R-squared	0.067	0.082	0.101	0.075	0.070	0.079

T statistics calculated based on White robust standard errors are in parentheses.

* Indicates 10% significance.

** Indicates 5% significance.

*** Indicates 1% significance.

For American social media, the statistical results of Table 5 shows that four presentation strategies played effective roles in developing the relationship between sojourners and Americans, but that there were differences in their degree of effectiveness. According to a ranking of their effect, the top three presentation strategies included two defensive ones, which were “reporting only good news” and “expressing controlled feelings,” and “reporting only good news” served as the most effective strategy to fulfill sojourners’ need for intercultural relatedness. This result was different from the situation on Chinese social media. That was, on Chinese social media, sojourners mainly adopted a proactive strategy to fulfill their need for relatedness with domestic relatives, friends, and Chinese Americans, while on American social media, sojourners preferred to use a defensive strategy to promote the fulfillment of their needs for relatedness. In the offline interview, the interviewees said that the strategy of “reporting only good news” could build a positive impression, activate dialog more quickly, and protect personal privacy. Such strategy conformed to the communication code of conduct on American social media, thus laying a good foundation for the establishment and maintenance of the interpersonal relations between Chinese sojourners and Americans. Additionally, the cultural context of American social media is obviously different from that of Chinese social media. In order to avoid possible cultural misunderstanding or even conflict, the interviewees said that they would control the limit of emotional expression on American social media. The results of interview analysis supported the quantitative research.

**Table 5:** The effect of different self-presentation strategies on the fulfillment of the need for relatedness in American social media.

Strategy choices:	Posting selected photos	Expressing humorous and close content	Displaying discipline	Reporting only good news	Expressing controlled feelings	Self-taunting
Strategy	0.014	0.162^**^	0.122^*^	0.174^***^	0.126^*^	0.088
	(0.23)	(2.13)	(1.70)	(2.96)	(1.80)	(1.26)
Gender	0.038	0.044	0.062	0.041	0.075	0.046
	(0.26)	(0.31)	(0.44)	(0.29)	(0.55)	(0.33)
Age	−0.016	−0.014	−0.017	−0.015	−0.016	−0.015
	(−1.33)	(−1.23)	(−1.37)	(−1.23)	(−1.34)	(−1.21)
Marriage	0.021	0.026	0.009	−0.022	−0.006	0.024
(married)	(0.11)	(0.14)	(0.05)	(−0.12)	(−0.03)	(0.13)
Education	−0.053	−0.069	−0.048	−0.054	−0.048	−0.056
	(−0.57)	(−0.77)	(−0.53)	(−0.62)	(−0.54)	(−0.63)
Years	0.032^**^	0.031^**^	0.033^**^	0.037^**^	0.034^**^	0.030^*^
	(2.00)	(2.01)	(2.11)	(2.26)	(2.26)	(1.89)
Online time	0.133^*^	0.115^*^	0.129^*^	0.119^*^	0.125^*^	0.122^*^
Per day	(1.79)	(1.67)	(1.86)	(1.76)	(1.79)	(1.72)
Constant	3.416^***^	2.954^***^	3.092^***^	2.911^***^	3.025^***^	3.192^***^
Term	(7.65)	(6.93)	(6.85)	(6.92)	(6.53)	(7.12)
Samples	223	223	223	223	223	223
R-squared	0.050	0.070	0.065	0.090	0.068	0.057

T statistics calculated based on White robust standard errors are in parentheses.

* Indicates 10% significance.

** Indicates 5% significance.

*** Indicates 1% significance.

## Discussion

Our study recruited 223 Chinese sojourners in the United States as research participants, investigated, and analyzed the relationship between their self-presentation behavior and the fulfillment of their psychological needs (autonomy and relatedness) on Chinese and American social media.

The study shows that, compared with American social media, the self-presentation behavior on Chinese social media can more significantly promote the fulfillment of sojourners’ need for autonomy. This paper holds that the main reason for this difference may be cultural context, that is, Chinese social media are more conducive to the realization of the sojourners’ autonomy. After all, there are cultural values and relational networks that the sojourners are familiar and identified with. The higher the degree of identification and integration with the cultural context, the higher the degree of autonomy of individual actions (Chirkov et al., 2003). In contrast, the cultural context of social media in the United States is relatively unfamiliar and features more heterogeneity. According to SDT, heterogeneity is a reverse force that hinders the realization of autonomy (Deci and Ryan, 1985, 2000); therefore, compared with the heterogeneous American social media, self-presentation behavior on Chinese social media is more active in promoting the satisfaction of the need for autonomy. Additionally, the results show that Chinese social media play a more active role in maintaining the relationship between sojourners and Chinese Americans than American social media. This result shows that the relatively homogeneous cultural context of Chinese social media provides sufficient emotional and spiritual exchange opportunities, as well as mutual social assistance space for sojourners and Chinese Americans, which is more recognized and adapted by both sides, thus helping to meet the fulfillment of their need for relatedness in the common cultural context (Lim and Pham, 2016; Xiao et al., 2018).

This study found that on Chinese social media, the comprehensive use of proactive and defensive presentation strategies helps to meet sojourners’ need for autonomy, which to a certain extent reflects the expediency of Chinese self-presentation behavior (Zhai, 2017, p. 56). That is, even when “expressing one’s true self,” sojourners still pay attention to what to say and what not to say, what kind of emotion needs to be expressed and what need not be, which generally reflects that sojourners are striking a balance between sense and sensibility on Chinese social media. At the same time, the sojourners not only distribute and adjust their presentation content but also pay attention to “voicing one’s opinion” through different forms of media, and Chinese social media is technically providing the sojourners with different kinds of effective ways to present ideal self-images and realize autonomous expression.

There are significant differences between Chinese and American social media in the use of self-presentation strategies that help to fulfill sojourners’ need for relatedness. On Chinese social media, a proactive strategy is more effective in meeting sojourners’ need for relatedness, while on American social media, sojourners tend to use a defensive strategy to promote the fulfillment of their need for relatedness. This paper argues that the differences in the connotation of the relationship between Chinese and American cultures affect sojourners’ tendencies when choosing presentation strategies. In the Chinese context, relationship (*guanxi*) is “a kind of social force exerted by family chain and social structure prior to individual existence” (Zhai, 2011, p. 187). Individuals must actively maintain important relationships for settling down and gain identification from the social environment at the same time. For Chinese sojourners, their intercultural identity and experiences more intangibly promoting them to adopt proactive presentation strategies on Chinese social media to meet their need for relatedness, because on the one hand, they can help them to consolidate different domestic relationships, and on the other hand, the maintenance of domestic relationships can provide them emotional attachment and a sense of belonging, which help them to alleviate various negative emotions caused by cultural maladjustment.

Compared with the *guanxi* in China, interpersonal relationships in the American context are clear “role relationships” and have a distinct public-private boundary (Chu, 1979). In the classic social interaction mode with an American-style interpersonal relationship at the core, the means of maintaining and developing the relationship presents very obvious characteristics of instrumental rationality (Altman and Taylor, 1973). Most of the Chinese sojourners who participated in this study came to the United States between 1 and 2 years prior. With the purpose of achieving their specific goals of sojourning in the United States, they needed to develop intercultural interpersonal relationships with local Americans as much as possible; on the other hand, the context of American social media is full of strangeness, heterogeneity, and uncertainty, which made the sojourners more cautious and more aware of all kinds of intercultural communication barriers. Therefore, based on the identification and understanding of the characteristics of relationships in an American context, Chinese sojourners are more likely to adopt a defensive strategy as the main and proactive strategy as the auxiliary to achieve the purpose of fulfilling their need for intercultural relatedness on American social media.

Unlike most previous studies that mainly analyzed the relationship between self-presentation strategies and psychological need fulfillment in a single cultural context, this paper provides empirical evidence for the first time on how self-presentation strategies affect fulfillment of psychological needs in the contexts of dual culture (host and home culture), which provides new inspiration for the study of online self-presentation behavior of sojourners, an important intercultural communication group.

## Future Directions

Future research might include empirical research on the relationship between online self-presentation strategies and the satisfaction of Chinese sojourners’ need for competence (Deci and Ryan, 2000) in the United States. In addition, future research might examine how the psychological effects of Chinese sojourners’ online self-presentation behavior affect their offline intercultural adaptation and communication, as well as the acquisition of social capital; such research should be strictly followed by an intercultural analysis of the causes of the general impact. On the basis of empirical research, future research might discuss ways to positively promote the intercultural adaptation and communication of international sojourners, and help sojourners to maintain their psychological well-being in host countries over the long run.

## Data Availability Statement

The original contributions presented in the study are included in the article/supplementary material, further inquiries can be directed to the corresponding author.

## Ethics Statement

Ethical review and approval was not required for this study on human participants, which was in accordance with local legislation and institutional requirements. The participants provided their written informed consent to participate in this study.

## Author Contributions

TY contributed to research design, theoretical discussion, and manuscript writing. QY contributed to data processing and empirical analysis. All authors contributed to the article and approved the submitted version.

### Conflict of Interest

The authors declare that the research was conducted in the absence of any commercial or financial relationships that could be construed as a potential conflict of interest.
